# Cardiac biomarkers in dialysis

**DOI:** 10.3934/genet.2017.1.1

**Published:** 2016-12-26

**Authors:** Usman Mahmood, David W Johnson, Magid A Fahim

**Affiliations:** 1 Department of Nephrology, Princess Alexandra Hospital, Australia; 2 Australasian Kidney Trials Network, School of Medicine, University of Queensland, Brisbane, Australia; 3 Translational Research Institute, Brisbane, Australia

**Keywords:** Biomarkers, Cardiovascular disease, Chronic kidney disease, Dialysis, Galectin, Hemodialysis, Natriuretic peptide, brain, N-terminal pro-B-type natriuretic peptide, Suppression of tumorigenicity 2, Troponin

## Abstract

Cardiovascular disease is the major cause of death, accounting for approximately 40 percent of all-cause mortality in patients receiving either hemodialysis or peritoneal dialysis. Cardiovascular risk stratification is an important aspect of managing dialysis patients as it enables early identification of high-risk patients, so therapeutic interventions can be optimized to lower cardiovascular morbidity and mortality. Biomarkers can detect early stages of cardiac injury so timely intervention can be provided. The B-type natriuretic peptides (Brain Natriuretic peptide [BNP] and N-terminal pro-B-type natriuretic peptide [NT-proBNP]) and troponins have been shown to predict mortality in dialysis patients. Suppression of tumorigenicity 2 (ST2) and galectin-3 are new emerging biomarkers in the field of heart failure in both the general and dialysis populations. This article aims to discuss the current evidence regarding cardiac biomarker use to diagnose myocardial injury and monitor the risk of major adverse cardiovascular events in patients undergoing dialysis.

## 1. Introduction

There is a huge burden of cardiovascular disease in patients undergoing hemodialysis with over 80% of patients having at least one cardiac diagnosis at baseline i.e. ischemic heart disease (IHD) (39%), congestive heart failure (40%), arrhythmia (31%), or other heart diseases (63%) [1],[2]. Cardiac disease-related mortality rates are reported to be 18.4 per 100 patient-years and account for approximately 30% of all deaths in this group, making cardiovascular disease the leading category of death after withdrawal from renal replacement therapy [3]. These rates represent a 10–30 folds increased risk of cardiovascular death compared with age-, gender- and diabetic status-matched cohorts from the general population and remain valid across diverse geographic regions [4]. Cardiovascular disease is also a major cause of morbidity, with hospitalization rates of 34.7–56 per 100 patient-years [1], making it the leading cause of hospitalization among patients on hemodialysis regardless of age.

The excessive burden of cardiovascular disease in populations with renal disease is due to the fact that cardiovascular disease is both an important cause and consequence of chronic kidney disease (CKD). There is a high prevalence of traditional cardiovascular risk factors, particularly diabetes mellitus and hypertension, in individuals with end-stage kidney disease (ESKD) compared with the general population [1],[2],[4],[5]. Moreover, ESKD is itself an independent risk factor for cardiovascular disease [6], mediated through a variety of pathophysiological mechanisms, including volume overload [7], sympathetic nervous system over activity [8], hyperparathyroidism [9] and oxidative stress [10].

Sudden cardiac death (SCD) is the single leading cause of death in dialysis populations [1] and is reported to be primarily related to underlying cardiomyopathy, rather than coronary artery disease [2],[11]. The ability to identify ESKD patients at high risk of cardiovascular disease, particularly SCD, is crucial to instituting timely intervention and improving clinical outcomes. Emerging cardiac biomarkers, such as B-type natriuretic peptides, troponin and other novel peptides, show considerable promise in this regard. For the purpose of this review, we define a cardiac biomarker as a serological marker reflecting myocardial end organ injury and associated with an altered risk of major adverse cardiovascular events (MACE).

## 2. B-type natriuretic peptides

B-type natriuretic peptide (BNP) is a member of the natriuretic peptide system, which additionally consists of the A-, C-, D- and V- type natriuretic peptides and urodilatin [12], although D- and V- natriuretic peptides are not endogenous to humans. BNP is primarily expressed and secreted by the heart, particularly the ventricular myocardium [13],[14]. However, it can be expressed by a number of extra-cardiac sites, including the brain, adrenal gland, kidney and lungs [15]. Transcription of the BNP gene is induced by a number of pathophysiological stimuli, including mechanical myocardial stretch [16],[17] (volume overload) or strain [18] (pressure overload), ischemia [19], pro-inflammatory cytokines, such as interleukin-1β and TNFα [20], α- and β- adrenergic agonists (sympathetic overactivity) [21], and vasoactive factors, such as endothelin-1 [17] and angiotensin-II [22]. It is important to note that these stimuli overlap significantly with the pathological milieu implicated in the genesis and progression of cardiomyopathy in the dialysis population [7],[8], positioning BNP as a potentially important biomarker of cardiac risk in this group.

### 2.1. Role of BNP/NT-proBNP as cardiac biomarkers in the general population

BNP and NT-proBNP have a number of proven clinical applications in the general population, including improving the accuracy of clinical assessment for diagnosing cardiac failure in patients presenting with dyspnea [23],[24] and estimating prognosis in patients with an established diagnosis of cardiac failure [25]. Over the last decade, 11 randomized controlled trials have examined the role of BNP/NT-proBNP monitoring in guiding heart failure therapy compared to specialist care alone [26]–[36].

A recent meta-analysis reported that BNP/NT-proBNP-guided heart failure pharmacotherapy resulted in significant reductions in mortality in patients under 75 years of age compared to specialist care alone (hazard ratio [HR] 0.62, 95% confidence interval [CI] 0.45–0.85) [37]. It also resulted in reductions in heart failure and cardiovascular hospitalization [37]. These findings have led to the recommendation that hormonal monitoring be incorporated into specialist care of heart failure patients [38].

### 2.2. Impact of kidney disease on serum BNP/NT-proBNP concentrations

Both peptides are partially excreted unchanged renally with a fractional renal excretion of 15–20% [39]. Consequently, the vast majority of patients with ESKD have markedly elevated levels of B-type natriuretic peptides, including those without a prior diagnosis of cardiac failure [40]. Using a cut-off value for NT-proBNP of 300 pg/mL (the concentration used to exclude heart failure with 98% certainty in non-dialysis patients presenting with dyspnea) [41], serum NT-proBNP concentrations in the dialysis population are elevated in the order of 3–55 times this diagnostic threshold [42]–[46] (Table 1). These concentrations far exceed the increment expected due to reduced renal clearance alone and therefore likely reflect the high burden of cardiomyopathy in the renal dialysis population [47].

Biologic or within-person variation of NT-pro-BNP in dialysis patients is an important consideration during interpretation of serial measurements of this cardiac biomarker. Fahim et al. [48] concluded that between-person variation of NT-proBNP was large and markedly greater than within-person variation, indicating that NT-proBNP testing might better be applied using a relative change strategy in this population. Serial NT-proBNP concentrations need to double or halve to confidently exclude change due to analytic and biologic variation alone.

The hemodialysis procedure has also been shown to affect serum concentrations of BNP and NT-proBNP, with low-flux hemodialysis resulting in 2–16% reductions in serum BNP concentrations and 0–12% increases in serum NT-proBNP concentrations [49]–[52]. This differential clearance is related to the differing molecular weights of BNP (3.5 kDa) and NT-proBNP (8.5 kDa). BNP is a smaller peptide and is cleared by low flux membranes whereas NT-proBNP is only cleared by high flux membranes. The increase in serum NT-proBNP concentration likely reflects the effect of hemoconcentration following hemodialysis and ultrafiltration, since BNP and NT-proBNP are secreted in equimolar amounts from the myocardium. In contrast, hemodialysis using a high-flux membrane reduces the serum concentrations of both BNP and NT-proBNP by 9–40% and 9.4–35%, respectively [49]–[53]. Hemodiafiltration results in even greater clearance of natriuretic peptides due to its considerably larger dialyser membrane pore size and increased convective clearances [54]. Consequently, measurement of pre-/post-hemodialysis serum B-type natriuretic peptide concentrations is of limited clinical value as observed changes reflect dialysis-related clearance rather than short-term changes in volume state or cardiac function. Indeed, several investigators have reported no correlation between changes in serum BNP or NT-proBNP concentration across a hemodialysis session and ultrafiltration volume, blood pressure change or weight change [54]–[56].

**Table 1.** Summary of the characteristics and utility of key cardiac biomarkers.

Biomarker	Distribution in dialysis population	Diagnostic utility	Monitoring utility
NT-proBNP	Exceeds upper limit of normal in >93%. Between person coefficient of variation 153% [48]	Limited—due to high between person variation and influenced by several pathophysiological factors (volume state, LV function, ischemia and inflammation)	Promising—Change in concentration directly associated with risk of non-fatal and fatal cardiovascular events—precise monitoring limits and interventions yet to be established
Troponin	Exceeds upper limit of normal in >90% even in absence of acute myocardial injury. Between person Coefficient of variation 83% [107]	Useful in acute care settings using serial measurements—a change of >33% excludes biological variation with 99% certainty	Promising—change in serial values associated with risk of mortality, precise monitoring limits and interventions yet to be established [142]
ST2	Unknown	Promising—levels correlate positively with peak CK in ACS [114]	Promising—Prognostic ability not influenced by renal function [117]. Adds prognostic value to NTpro-BNP and cardiac troponins [116]
GAL-3	Unknown	Limited	Promising—independent predictive value for mortality in chronic heart failure [122]

In contrast to hemodialysis, the effect of peritoneal dialysis on the clearance of plasma BNP and NT-proBNP remains unclear. A recent study of eight peritoneal dialysis patients demonstrated that BNP was present in peritoneal dialysis fluid at mean concentration of 19 ± 4% that of circulating BNP concentrations, suggesting that BNP is cleared by peritoneal dialysis [57]. Whether the same holds true for the larger molecular weight peptide, NT-proBNP, was not investigated, nor was the effect of transporter status and/or ultrafiltration volume on clearance kinetics. In contrast, a study of 11 patients on nocturnal intermittent peritoneal dialysis (NIPD) investigating the effect of a dialysis session on plasma concentrations of BNP/NT-proBNP found no change in circulating concentrations of either peptide across the procedure. Indeed, there was also no difference in concentrations during or between dialysis procedures [58]. The discordance between these studies may reflect the small sample sizes used. The clearance of BNP/NT-proBNP by peritoneal dialysis warrants further investigation to aid the interpretation of serial natriuretic peptide measurements.

### 2.3. B-type natriuretic peptides as markers of volume state in dialysis

BNP/NT-proBNP concentrations have not been shown to correlate with short-term changes in volume state [55],[56]. Their role as volume markers in the medium to long-term remains an important, unresolved issue. Some studies have found no association between circulating levels of these peptides and volume state assessed by clinical examination [59] or bioimpedance [60], although seven larger cross-sectional studies have reported significant direct correlations between serum BNP/NT-proBNP concentrations and volume state assessed objectively by bioimpedance analysis [61]–[65], or a combination of clinical assessment, echocardiography and/or chest X-ray findings [66],[67]. Thus, the bulk of available evidence suggests that circulating B-type natriuretic peptide concentrations reflect, at least in part, volume state in ESKD over the medium-to-long term. Answering this question definitively will require a longitudinal study with an adequate sample to accurately and repeatedly assess the dynamic relationship between volume state and natriuretic peptide concentrations after controlling for other confounders.

### 2.4. B-type natriuretic peptides as biomarkers of cardiomyopathy in ESKD

Several studies in dialysis populations have demonstrated that BNP/NT-proBNP have an independent and powerful direct correlation with left ventricular (LV) mass [68]–[71], and with cardiac diastolic [72],[73] and systolic dysfunction [43],[46],[68],[70]. A multivariable analysis of the predictors of cardiomyopathy in a cohort of 246 prevalent hemodialysis and peritoneal dialysis patients found that log-transformed serum BNP concentration was a significant predictor of LV ejection fraction (*β* = −0.48, *p* < 0.0001) and LV mass indexed to height (*β* = 0.36, *p* < 0.0001) after adjusting for blood pressure, age, diabetes, albumin, hemoglobin, treatment modality, small molecule clearance, and time on renal replacement therapy [68]. Similarly, in a cross-sectional study of 99 hemodialysis patients with preserved systolic function, investigators reported significantly higher BNP concentrations in individuals with diastolic dysfunction compared to those without cardiac dysfunction (314 ± 162 ng/L vs 131 ± 77 ng/L, *p* = 0.01). A cohort study of 199 dialysis patients investigated the relationship between baseline BNP as the exposure and increase in left atrial volume as the outcome. The study demonstrated that baseline BNP concentration was a powerful, independent predictor of increase in left atrial volume [74]. Finally, a longitudinal study of 21 hemodialysis patients undergoing serial measurement of NT-proBNP and LV mass at baseline, 6- and 12- months demonstrated a powerful direct correlation between change in NT-proBNP concentration and change in LV mass (r = 0.78, *p* < 0.001) [71].

### 2.5. B-type natriuretic peptides as prognostic biomarkers in ESKD

In addition to the important cardiac and physiological correlations discussed previously, cohort studies using a single baseline serum concentration of BNP/NT-proBNP as the exposure have demonstrated that it is a powerful, independent predictor of heart failure [43],[75], sudden cardiac death [11], and both cardiovascular [42]–[45],[68],[76] and all-cause mortality [42]–[45],[68] in ESKD. For example, Wang et al. [43] demonstrated that serum NT-pro-BNP concentration was an important risk predictor of cardiovascular congestion, mortality, and adverse cardiovascular outcomes in a cohort study of 230 prevalent peritoneal dialysis patients followed for 3 years.

The strongest support for the potential role of the B-type natriuretic peptides as biomarkers of cardiac risk in the renal dialysis population comes from longitudinal studies employing limited numbers of repeated natriuretic peptide measurements to investigate the relationship between change in peptide concentration and change in risk of mortal events. These studies demonstrated that change in B-type natriuretic peptide concentration correlated directly with change in risk of cardiovascular and all-cause mortality in both incident and prevalent hemodialysis patients [42],[76],[77]. While these studies are an important first step in delineating the clinical application of this biomarker in dialysis, they are limited by their paucity of repeated measures, and lack of time to event and accuracy measures (sensitivity and specificity); all of which are essential components to advance this promising marker into clinical practice [78].

## 3. Cardiac troponins

The troponin complex is composed of three subunits—Troponin C (TnC), Troponin I (TnI), and Troponin T (TnT)—which work in concert to regulate the interaction between actin and myosin required for myofibrillary contraction. Approximately 6% of cTnT and 4% of cTnI are present unbound in the cytoplasm of cardiac myofibrils [79] and can be released rapidly into the circulation following ischemia [80] (with or without necrosis) or in response to myofibrillary stretch/strain, which result in an increase in cell membrane permeability [81]. The remainders of cTnI and cTnT in cardiac myocytes are present in the form of the ternary troponin complex bound to actin and tropomysosin and are released into the circulation when cardiac myofibrils undergo necrosis following ischemia, inflammation, infiltration or trauma. Although evaluation of serum concentrations of troponins for diagnosis of acute coronary syndromes is standard practice in non-renal populations [82], their interpretation in patients with ESKD is more problematic. Several issues continue to confound the interpretation of cardiac troponins in both the acute and chronic settings in ESKD populations, including a lack of clarity regarding what constitutes a significant change in serial measurements, disagreement regarding the pathophysiological associations of troponins, and whether interpretation in chronic settings is best undertaken using single or serial measurements.

### 3.1. Impact of kidney disease on serum troponin concentrations

Troponin is one of the biologically active structural proteins released into the circulation when cardiac injury occurs with disruption of normal cardiac myocyte membrane integrity. The precise mechanism by which cardiac troponins are cleared from the circulation has not been elucidated, although accumulating evidence suggests that renal clearance plays little, if any, role in the elimination of troponins. Firstly, several cohort studies have demonstrated that a large proportion of renal transplant recipients with elevated pre-transplant cardiac troponin concentrations continue to have elevated concentrations in the first year post-transplant despite a substantial improvement in their glomerular filtration rate [83]–[86]. Secondly, the half-life of cardiac troponins is not significantly different between patients with and those without chronic kidney disease [87]. Finally, the only circulating form of cTnT measurable in asymptomatic dialysis patients with elevated cTnT concentrations is the intact peptide, which has a molecular weight of 37 kDa thereby making it too large for glomerular filtration [88].

Nevertheless, a large proportion of dialysis patients have elevated serum cardiac troponin concentrations, which persistently exceed the 99^th^ centile upper reference limit of troponin assays despite being asymptomatic (Table 1) [89]–[93]. The prevalence of this finding varies according to the type of cardiac troponin measured and the sensitivity/generation of the assay employed. For cTnT, between 29–43% of patients have been reported to have elevated concentrations using first generation TnT assays [94],[95], as compared to 99% of dialysis patients when the latest, fifth generation high-sensitivity assays are employed [93]. Observational studies have also consistently demonstrated that a higher proportion of dialysis patients have an abnormal cTnT concentration compared with cTnI when both cardiac troponins are measured simultaneously within the same dialysis cohort [96]–[98]. The precise reasons for the discordance are unknown but may be related to uremia-associated modifications of circulating cTnI, which interfere with measurement, adsorption of cTnI to hemodialysis dialyser membranes [99], and/or anti-cTnI antibodies, which interfere with, assay performance [100]. cTnT and cTnI are partially cleared during hemodialysis with high flux dialysis membranes, but not with low flux membranes [93],[98]. This has important implications for the interpretation of serial troponin measurements.

### 3.2. Interpretation of troponin measurements in dialysis patients in acute care settings

Patients on maintenance dialysis have a high incidence of acute coronary syndromes (9.8–16 per 100 patient-years), which are a leading cause of emergency presentations, hospitalization and death in this population [1],[43],[101],[102]. Accurately diagnosing acute coronary syndromes in dialysis patients is challenging due to the high proportion of patients who present with atypical symptoms and electrocardiographic changes [103],[104]. This has led to greater reliance on biomarkers of myocardial injury in this population. However, the interpretation of cardiac troponins from dialysis patients in acute settings is not straightforward given that the majority has cardiac troponin concentrations that persistently exceed the 99^th^ centile upper reference limit of the assay used even when they are well; reflecting established rather than acute cardiovascular pathology [89]–[93].

In an effort to address this conundrum, the current consensus guideline on the diagnosis of acute myocardial injury requires demonstration of a *change in serial troponin concentrations* with at least one concentration exceeding the 99^th^ centile upper reference limit, occurring in an appropriate clinical context, in order to confirm a diagnosis of acute myocardial injury in dialysis patients [82]. Unfortunately, this recommendation is not accompanied by any evidence-based guidance on precisely how much change in serial measurements constitutes a significant change that can discriminate between biological variation and acute myocardial injury leading to considerable diagnostic confusion [105]. Guidelines have either provided no guidance on the magnitude of change in serial troponins that excludes biological variation in dialysis patients [82],[105] or have based their guidance on the analytic performance of troponin assays rather than clinical studies, wherein a 20% magnitude of change has been recommended based on the fact that such a delta equates to 3 standard deviations of the troponin assay's analytic variation [106].

Guidelines for interpreting serial troponin measurements should incorporate data on both the biological variation of cardiac troponins in stable dialysis patients and serial changes in troponins associated with adverse outcomes in dialysis patients. Biological or within-person variation describes the random fluctuation of biomarker levels around a homeostatic set point in healthy individuals or those with stable disease; such fluctuations are of no clinical significance and must be distinguished from pathological changes. Fahim et al. [107] concluded that between-person variation of hs-cTnT in the dialysis population was markedly greater than within-person variation indicating that hs-cTnT testing is best applied in this population using a relative change strategy. An increase of 33% or a reduction of 25% in serial serum hs-cTnT concentrations measured at weekly intervals excludes change due to analytical and biological variation alone with 99% confidence. This study enrolled both hemodialysis and peritoneal dialysis patients and ensured the stability of important covariates, including dialysis and pharmacological prescriptions, cardiac rhythm, and patient volume status, while concurrently excluded patients who experienced an adverse cardiovascular outcome from the study's analysis.

### 3.3. Interpretation of cardiac troponins in dialysis patients in chronic care settings

Michos et al. [108] have also demonstrated an association between chronically elevated cardiac troponin concentrations and adverse outcomes in dialysis patients in the absence of acute symptoms, implying that cardiac troponins may have a role as prognostic and/or monitoring biomarkers. Moreover, a recent systematic review and meta-analysis of 98 cohort studies found that serum cTnT and cTnI concentrations exceeding the assay's upper reference limit were associated with increased risks of all-cause mortality (HR = 3.00 [95% CI 2.36 – 4.26] and HR = 2.70 [95% CI 1.90 – 4.57], respectively), and cardiovascular mortality (HR = 3.31 [95% CI 1.81 – 5.53] and HR = 4.20 [95% CI 2.01 – 9.20], respectively) among asymptomatic dialysis patients even after adjustment for age and presence of cardiovascular co-morbidity [108]. These results suggest that monitoring troponin concentrations may have a role in identifying patients at increased risk of adverse cardiovascular events and have led to the licensing of cardiac troponins by the United States Food and Drug Administration (FDA) for prognostication in this population. However, despite this regulatory approval, troponins have failed to enter routine clinical practice due to ambiguity regarding the ideal strategy for testing and interpreting cardiac troponins, and the resultant action that should be taken based on testing [109]. In order to advance troponin testing in clinical dialysis practice, longitudinal cohort studies are needed to determine whether the test is best interpreted using an absolute cut-off or relative change between serial measurements, the frequency of measurements, the time to event between an abnormal test and an adverse outcome, and the targets of therapy.

## 4. B-type natriuretic peptides and Cardiac Troponins: ready for prime time?

Based on the evidence presented and discussed in this review, troponin has been validated as a useful biomarker in the acute care setting and should be used to diagnose myocardial injury in ESKD patients using a relative change strategy. Consequently, in an ESKD patient with a compatible clinical history, an acute increase in serial serum hs-cTnT concentrations of greater than 33% is sufficient to diagnose acute myocardial injury. On the other hand, we do not recommend the use of cardiac troponin in the chronic care setting in these patients. Chronically elevated cardiac troponin concentrations in clinically well dialysis patients reflect long term risk of mortality, but are not well characterized enough for use in clinical practice. Elevated troponin concentrations in dialysis patients have been repeatedly shown to predict risk of future adverse cardiovascular events, however, the precise magnitude of change that should prompt action, the interventions that should be instituted, and the benefits of such monitoring have not been studied precluding a recommendation for use in routine practice. Similarly, whilst B-type natriuretic peptides should be used to guide heart failure therapy, we do not recommend their use in acute or chronic care settings to diagnose myocardial injury or damage in ESKD patients. In contrast to the use of NT-proBNP in the non-dialysis population, it is unlikely that a threshold value of NT-proBNP can be derived for either diagnosing or excluding structural cardiac pathology in dialysis population. This is due to the large between person variations of NT-proBNP in dialysis population. A clinically useful strategy requires precise guidance on a magnitude of relative change in serial NT-proBNP concentrations that can safely be ignored (biological variation) versus that should prompt action, the associated time to event of such a change, the ideal monitoring interval, clear targets for intervention, and evidence that such intervention improves patient level outcomes. These parameters have yet to be established.

## 5. Emerging cardiac biomarkers

Suppression of tumorigenicity 2 (ST2) and galectin-3 (GAL-3) are emerging biomarkers in the field of heart failure (Table 1). They seem to have additional but limited prognostic values on top of the clinical models and other more established biomarkers (i.e., NT-proBNP and troponin). The 2013 American College of Cardiology/American Heart Association guidelines for the management of heart failure mentioned both galectin-3 and ST2 as emerging biomarkers that are not only predictive of hospitalization and death in patients with heart failure, but also add additional prognostic value over natriuretic peptides [110]. Both of these biomarkers reflect tissue damage, independent of cardiac loading conditions and may supplement the currently used markers for heart failure. Additionally, increased ST2 and galectin-3 concentrations have been reported in association with inflammatory disease, such as pneumonia and chronic obstructive pulmonary disease [111].

### 5.1. ST2

ST2 is a member of Toll-like/IL-1 receptor superfamily with transmembrane (ST2L) and soluble isoforms (sST2). It was discovered in 1989 and its downstream effects include activation of T-helper type 2 (Th2) cells and production of Th2-associated cytokines. sST2 binds to IL-33 and functions as a ‘decoy’ receptor for IL-33, thereby attenuating the systemic effects of IL-33 [112]. sST2 concentration is increased in inflammatory and heart diseases, and has emerged as a clinically useful prognostic biomarker in patients with cardiovascular disease and acute dyspnea [112],[113]. ST2 expression is upregulated after myocardial ischemia or mechanical stress, and plays a role in cardiac remodeling after ischemic injury, which makes it an attractive biomarker to predict future clinical HF in patients with myocardial ischemia [114]. Weinberg et al. [114] also showed increased sST2 levels in patients after myocardial infarction, with values correlating positively with peak creatine kinase (CK) and negatively with left ventricular ejection fraction (LVEF). Similarly, Demyanets et al. [115] found that sST2 levels were significantly increased in patients with acute coronary syndrome (ACS) as compared to patients with stable coronary artery disease and control individuals without significant stenosis on coronary angiography. Moreover, Dieplinger et al. [116] showed that sST2 added prognostic value to the well-established cardiac biomarkers, NT-proBNP and cardiac troponin. Patients at low, intermediate and high risks of all-cause and cardiovascular mortality were also identified by using a simple multi-biomarker approach, which combined ST-2, NT-proBNP, and high sensitivity (hs) cardiac troponin T (cTnT) [116].

The relationship of ST2 and renal function (measured by eGFR) was investigated in a Spanish study of 891 consecutive patients treated at a structured multidisciplinary heart failure unit [117]. This study demonstrated that the prognostic value of ST2 was not influenced by renal function, suggesting that ST2 may be advocated as a preferable biomarker in patients with renal insufficiency, a comorbidity that is very common in heart failure and is among the strongest predictors of adverse outcomes. They concluded that in a “real-life” cohort of heart failure patients, the addition of ST2 and NTproBNP substantially improved the risk stratification for death beyond that of a model that was solely based on established mortality risk factors, including renal impairment.

### 5.2. Galectin-3 (GAL-3)

GAL-3 is a marker of tissue fibrosis, which is a hallmark of cardiac remodeling and heart failure. This can be reliably measured in the blood and studies have shown its prognostic value in heart failure [118]. GAL-3 is a 31-kDa member of family of lectins that bind beta-galactosides by either N-linked or O-linked glycosylation through their carbohydrate recognition domain (CRD) [119]. They have been numbered according to their order of discovery (i.e., from GAL-1 to GAL-15). Sharma et al. demonstrated that GAL-3 was overexpressed by macrophages at an early stage of myocardial dysfunction, even before the onset of heart failure, and that continuous infusion of recombinant GAL-3 in mice triggered cardiac fibroblast proliferation and collagen deposition, thereby ultimately leading to ventricular dysfunction [120]. Several studies of plasma GAL-3 as a biomarker in heart failure have been published [121]. A recent meta-analysis by Chen et al. [122] showed that GAL-3 was independently predictive of mortality in chronic heart failure. Moreover, Winter et al. [123] concluded that elevated levels of circulating GAL-3 were strongly associated with premature myocardial infarction and that GAL-3 might serve as a link between dyslipidemia (as a driving force of plaque formation) and inflammation (as an initiator of plaque rupture) in patients with premature acute myocardial infarction. GAL-3 has been shown to be the best short-term predictor of events in patients with heart failure, which led to incorporation of this measurement in the current American Heart Association guidelines Heart Failure guidelines for risk stratification purposes of such patients [124]. Hogas et al. [125] recently demonstrated that similar to the general population, GAL-3 is an independent predictor of mortality in HD patients. Gurel et al. [126] concluded that galectin-3 might be a promising biomarker for the detection of left ventricular diastolic dysfunction in patients undergoing maintenance hemodialysis. GAL-3 has the potential as a new modifiable risk factor for heart failure. Currently, attempts are being made to target or inhibit galectin-3 using natural or pharmaceutical inhibitors with the aim to ameliorate heart failure. Available experimental evidence suggests that GAL-3 inhibition indeed may represent a novel tool to treat heart failure [127].

## 6. Other novel cardiac biomarkers

Growth differentiation factor-15 (GDF-15) is a 12-kDa secreted protein belonging to transforming growth factor-beta cytokine family. Hagstrom et al. [128] measured GDF-15 in 16,876 patients, and showed that it was independently associated with an increased risk of spontaneous myocardial infarction. Moreover, the GDF-15 level in patients with acute coronary syndrome (ACS) was found to be independently associated with an enhanced risk of stroke and strongly correlated with an increased risk of cardiovascular disease and total mortality in ACS patients [128]. As an emerging biomarker, GDF-15 shows promise because it was found to be increased in early subclinical disease, retaining prognostic utility for cardiovascular disease events and mortality.

Adrenomedullin (ADM) is released from a multitude of tissues and was first isolated from human pheochromocytoma cells. It has potent vasodilatory, hypotensive and natriuretic properties [129] mediated by cyclic adenosine monophosphate (cAMP), nitric oxide and renal prostaglandin systems. Its concentration is elevated in chronic heart failure and it is increased in proportion to disease severity [130]. Its clinical use has been limited by lack of stability *in vitro*. Measurement of the inactive, stable, mid-regional (MR) prohormone of this peptide (MR-proADM) is now possible. Maisel et al. [131] (Biomarkers in Acute Heart Failure (BACH) trial) found that MR-proADM was superior to BNP or NT-proBNP in identifying dyspneic patients with acute decompensated HF at high risk of 90-day mortality.

Copeptin, a glycoprotein, is a marker of neurohormonal activation and part of the prehormone molecule of the antidiuretic hormone or arginine vasopressin. There is evidence that copeptin might be useful as a diagnostic or prognostic biomarker and risk-stratifier in a range of cardiovascular disease conditions [132].

Osteoprotegerin (OPG) has emerged as independent biomarker of cardiovascular disease in patients with acute or chronic heart disease as well as in the healthy population. OPG is a mediator of vascular calcification [133], which is a part of the atherosclerotic process leading to clinical cardiovascular disease. OPG levels have been positively correlated with coronary calcification, vascular stiffness and the presence of unstable atherosclerotic plaques [134]. OPG contains a heparin binding domain and studies have shown that it is rapidly released from smooth muscle cells after heparin treatment. Therefore, the timing of blood sample collection in relation to hemodialysis (which generally involves heparin administration) is important and can make it difficult to compare studies of OPG levels in patients with acute CV events.

Osteopontin is a glycoprotein that is expressed in various cell types, including cardiomyocytes and fibroblasts and has been shown to be significantly increased in patients with heart failure irrespective of the underlying cause and correlate with the severity of heart failure [135].

Adiponectin is a multifunctional adipocyte-derived protein with anti-inflammatory, antiatherogenic and insulin sensitizing activity [136]. CKD is associated with hyperadiponectinemia including nephrotic syndrome [137]. Hypoadiponectinemia predicts the development of new cardiovascular events in patients with CKD, including the hemodialysis population. Zoccali et al. showed in their cohort of 227 hemodialysis patients followed for a mean period of 31 ± 13 months that each 1 µg/mL increase in serum adiponectin concentration was associated with a 3% risk reduction in new cardiovascular events [138].

Matrix metalloproteinase 9 (MMP-9) is a potential biomarker for cardiac remodeling. MMP-9 levels have been shown to correlate with LV enlargement, lower ventricular ejection fraction, and persistent adverse LV remodeling in chronic systolic heart failure patients [139].

Fetuin-A is predominantly made by the liver and is decreased in heart failure patients, indicating that anti-inflammatory activity is down regulated in these patients and that calcification may be associated with heart failure [140].

Although all of these very novel biomarkers have diagnostic and prognostic potential, their clinical utility remains unclear currently.

## 7. Conclusion

Dialysis patients have up to 100-fold increased risk of cardiac death compared to the general population. This increased risk has remained unchanged over the last decade, whilst patient numbers on dialysis have grown exponentially. A key factor underpinning this mortality risk is fluid overload-induced cardiomyopathy. Progress in improving outcomes has been limited by our current inability to assess fluid state and cardiac risk status in an accurate and dynamic manner. NT-proBNP is a promising biomarker that has been shown in preliminary studies to predict mortality and correlate with cardiomyopathy and fluid state. The two cardiac troponins, cTnI and cTnT, represent potentially valuable biomarkers for the diagnosis and management of cardiovascular disease in dialysis patients. Future randomized studies should investigate if early intervention for minor, but significant, changes in cardiac troponin concentrations improves patient outcomes in the dialysis population as has been demonstrated in the non-dialysis population [141]. Both ST2 and galectin-3 have shown prognostic value in patients with acute and chronic heart failure on top of natriuretic peptides in general population. Strategies that combine multiple biomarkers may ultimately be the gold standard in guiding heart failure therapy in the future. Appropriately designed and followed-up studies with larger sample sizes, that incorporate biomarker based treatment algorithms in dialysis population need to be performed to determine if this approach is useful and economical in the long run. Learning to use these biomarkers efficiently and to assess and risk stratify patients with cardiovascular disease to provide tailored therapeutic strategy should be the priority of today's clinicians.
